# Corneal Viscoelastic Properties from Finite-Element Analysis of *In Vivo* Air-Puff Deformation

**DOI:** 10.1371/journal.pone.0104904

**Published:** 2014-08-14

**Authors:** Sabine Kling, Nandor Bekesi, Carlos Dorronsoro, Daniel Pascual, Susana Marcos

**Affiliations:** Instituto de Óptica “Daza de Valdés”, Madrid, Spain; Cardiff University, United Kingdom

## Abstract

Biomechanical properties are an excellent health marker of biological tissues, however they are challenging to be measured in-vivo. Non-invasive approaches to assess tissue biomechanics have been suggested, but there is a clear need for more accurate techniques for diagnosis, surgical guidance and treatment evaluation. Recently air-puff systems have been developed to study the dynamic tissue response, nevertheless the experimental geometrical observations lack from an analysis that addresses specifically the inherent dynamic properties. In this study a viscoelastic finite element model was built that predicts the experimental corneal deformation response to an air-puff for different conditions. A sensitivity analysis reveals significant contributions to corneal deformation of intraocular pressure and corneal thickness, besides corneal biomechanical properties. The results show the capability of dynamic imaging to reveal inherent biomechanical properties in vivo. Estimates of corneal biomechanical parameters will contribute to the basic understanding of corneal structure, shape and integrity and increase the predictability of corneal surgery.

## Introduction

The demand for measuring biomechanical properties of biological tissue in-vivo and non-invasively is high, because abnormal tissue biomechanics play a key role in a wide range of diseases. The stress distribution [1] around and stiffness [2] of tumor tissue largely determine its progression. Biomechanical properties are also indicative of muscle function [3] and the effects of disease, wound healing [4], aging or cosmetics [5].

In ophthalmology, ocular biomechanics are essential for basic research, clinical evaluation, prognosis and treatment. Pathological weakening of the cornea appears to be responsible for the corneal bulging, and dramatic visual degradation in keratoconus. Corneal collagen cross-linking is an emerging treatment to increase corneal stiffness in this disease [6]. Theoretical models that integrate individual mechanical, geometrical and structural patient data have the potential to improve clinical outcomes of eye surgery, but depend largely on the identification of pre-operative biomechanical parameters. Most frequently only the elastic tissue properties are evaluated, more specifically the elasticity modulus. However, also time-dependent mechanical properties are expected to matter, along with active remodeling processes. For example, the progressive deformation of the cornea (ectasia) occurring in keratoconus [17] and after some laser refractive procedures [18], may result from an altered stress distribution of the cornea inducing viscoelastic deformation until the new steady state is reached. Also certain treatments such as UV corneal collagen cross-linking (CXL) likely modify both elastic and viscoelastic properties. Changes in the degree of collagen interweaving, keratocyte density or the presence of hydrophilic proteoglycans may result in the viscoelastic failure or abnormal repair [19].

Today, most information regarding available corneal biomechanical properties was assessed ex vivo [7], [8], [9], [10], where changes in the hydration state [9] and other non-physiological conditions affect the measurement.

In vivo approaches to measure corneal biomechanical properties include stepwise indentation with a cantilever [11]; ultrasonic [12] and magnetic resonance [13] techniques; corneal optical coherence elastography [14]; phase-sensitive [15] Optical Coherence Tomography (OCT); and Brillouin microscopy [16]. Drawbacks of several of these techniques include that they only can be operated at low speed, have a low spatial resolution or require contact with the patient’s corneal surface.

Studying the dynamic deformation following an air-puff has recently been proposed in different biomedical areas (skin [5], bacteria [20], cornea [21], soft tissue tumors [22]) to non-invasively assess biomechanical properties, but also in other fields to study chicken embryogenesis [23], fruit firmness [24] or meat tenderness [25]. In most cases the degree of deformation of the sample is empirically related to mechanical parameters, and the inherent mechanical parameters of the tissue were rarely retrieved. To our knowledge, only Boyer et al [5] proposed an analytical estimation of the “restricted Young's modulus” from experimental deformation curves in skin.

Air puff applanation of the cornea is a frequent technique in ophthalmology to measure intraocular pressure, yet requiring correction formulae to account for corneal thickness and stiffness. Recently, high speed Optical Coherence Tomography [26] and Scheimpflug imaging systems [27] have been proposed allowing dynamic imaging of corneal cross-sections during the air-puff deformation event. Experimental studies confirm that the spatio-temporal corneal deformation pattern depends on the inherent mechanical properties, as well as on corneal geometry and intraocular pressure [21].

Some studies have used forward biomechanical modeling of the corneal tissue to predict the corneal response to incisional surgery [28] or laser ablation [29]. On the other hand, inverse modeling is used to retrieve inherent material properties matching the response of the model to the experimental response. Previous studies have obtained elastic properties from inverse modeling of topographic differences before and after refractive surgery [30]. Also the anisotropic properties of corneal tissue have been determined from inverse modeling of corneal inflation experiments [31].

In this study we present a finite element model that predicts the corneal deformation pattern upon air-puff ejection and which has allowed, for the first time to our knowledge, to retrieve both, the elastic and viscoelastic properties of the cornea. The model was validated with experimental data of porcine and human eyes and the sensitivity of corneal deformation to geometrical and biomechanical parameters was studied. Being able to retrieve dynamic material properties will open a new way for tissue characterization in vivo.

## Methods

**Figure 1:**
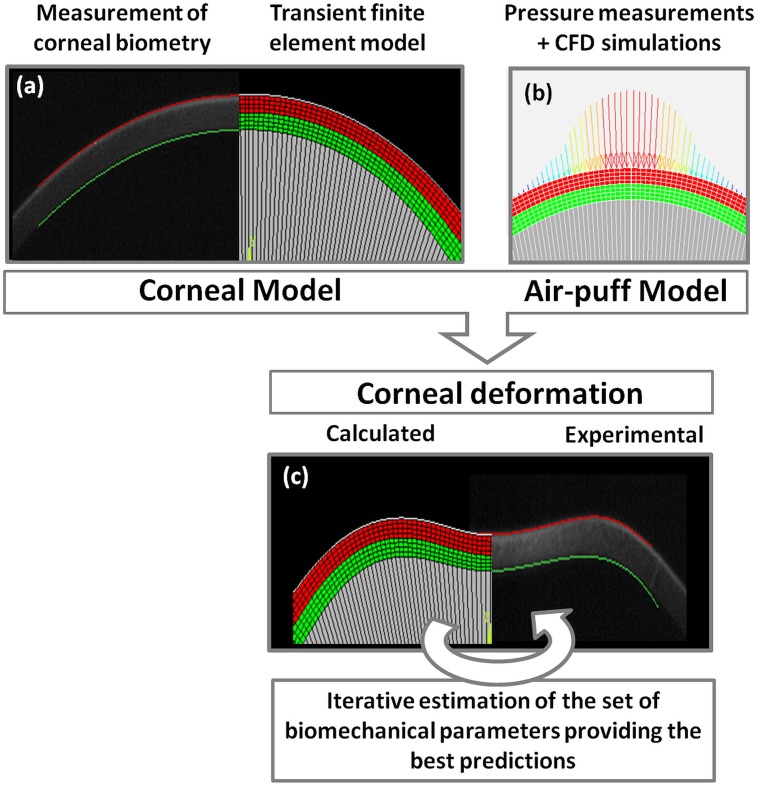
Schematic illustration of the simulation procedure. (a) Corneal geometrical data from Scheimpflug images are used to define the model geometry. Inverse modeling is performed to account for the effect of applying the IOP. (b) The temporal pressure profile measured experimentally and the spatial pressure profile obtained from CFD (Computation Fluid Dynamics) simulation are applied to the cornea as a function of time, location and current deformed shape. (c) The finite element model is solved for the current parameter set and simulation results are compared to the experimentally measured deformation. A step-wise optimization approach is used to find the parameter set that leads to the most similar deformation.

Corneal deformation was studied using a finite element model with a two-dimensional axis-symmetric geometry. Initial corneal curvature, thickness dimensions and the dynamic deformation response were available from previous experimental Scheimpflug cross-sectional images of the anterior eye segment (see Figure 1). To simulate the experimental conditions accordingly, the pressure characteristics provided by the air-puff system were determined: First, the temporal pressure profile was measured experimentally and then a computational fluid dynamics simulation was performed to determine the spatial pressure profile. Inverse modeling was performed in order to find the biomechanical parameter set that best represented the different experimental conditions. Finally a sensitivity analysis was performed in order to determine the parameters that were correlated the most to the corneal deformation response following an air-puff.

### Air Puff Imaging

The Corvis system (Oculus, Wetzlar, Germany) uses high-speed Scheimpflug imaging to capture the spatio-temporal corneal deformation following an air-puff. Typically 140 images are taken during the ∼30 ms deformation event (i.e. at a speed of about 4330 images/sec) with a resolution of 640×480 pixels. The spatial and temporal deformation profiles are highly reproducible (RMS difference 0.015 mm and 0.012 mm, respectively).

### Experimental data

Experimental corneal deformation data following an air-puff were taken from a previous study in porcine eyes ex vivo and human eyes in vivo and ex vivo [21]. Porcine eyes were obtained from a local slaughterhouse (Patel S.A.U., Barcelona, Spain). Human donor eyes for explicit therapeutic or scientific use were obtained through a collaborative agreement with Universidad Autónoma de Madrid and Transplant Services Foundation (Banco de Sangre y Tejidos, Barcelona, Spain, http://www.bancsang.net) and used within less than 12 hours *post mortem*. Human subjects were normal patients (35.4 years of age on average) and signed an informed consent after receiving an explanation regarding the nature of the study. All protocols are in accordance with the tenets of the Declaration of Helsinki and had been approved by the Institutional Review Boards (CSIC Ethics Committee, Bioethics Subcommittee, Madrid, Spain). Ex vivo porcine corneas were measured under different hydration (after photosensitizer 0.125%-riboflavin-20%-dextran instillation), stiffness (after UV collagen cross-linking), boundary conditions (corneal button, eye globe ex vivo, eye globe in vivo) and intraocular pressures (IOP) in order to evaluate the effect of the different parameters on the corneal deformation pattern. For this study we used a subset of these experimental data: (1) ex vivo cross-linked pig eye globes (n = 5), (2) ex vivo human eye globes (n = 5); and (3) human eyes in vivo (n = 9). Corneal experimental input parameters include the un-deformed corneal geometry, the temporal profile of corneal apex indentation, and the spatial corneal deformation pattern. Average deformation values of each condition were used as input to the simulation.

### Temporal air-puff characterization

A pressure sensor (MPX2301DT1, Freescale Seminconductor Inc, Tempe, AZ, USA) was used to measure the central temporal pressure distribution of the air-puff at 11-mm distance (typical position of the cornea) from the air-tube. The temporal pressure profile was fit by a linear function for pressure increase (slope = 5.38 mmHg/ms; r = 0.9883; p<0.001) as well as for pressure decrease (slope  = −8.38 mmHg/ms; r = 0.9897; p = 0.03). Although the overall air-puff duration was 27.50 ms, only 20.63 ms (i.e. 97% of the air-pressure) contributed effectively to the corneal deformation.

### Spatial air-puff characterization

In order to determine the geometry-dependent spatial shape of the air-puff at different stages of the corneal deformation, the maximum air speed (115 m/s) was estimated by Bernoulli’s equation at the stagnation point of the airflow:
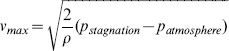
(1)where ρ is density, p_stagnation_ is the measured absolute stagnation pressure of the air puff, and p_atmosphere_ is the static atmospheric pressure. This value is comparable with the air speed measured previously [32] using a hot wire anemometer next to the tube end (>100 m/s).

To retrieve the spatial air pressure distribution, an axis-symmetric computational fluid dynamics (CFD) simulation was performed using the finite volume method (FVM) implemented in the Fluent module of the ANSYS Release 14.0 software package.

#### CFD Geometry

**Figure 2:**
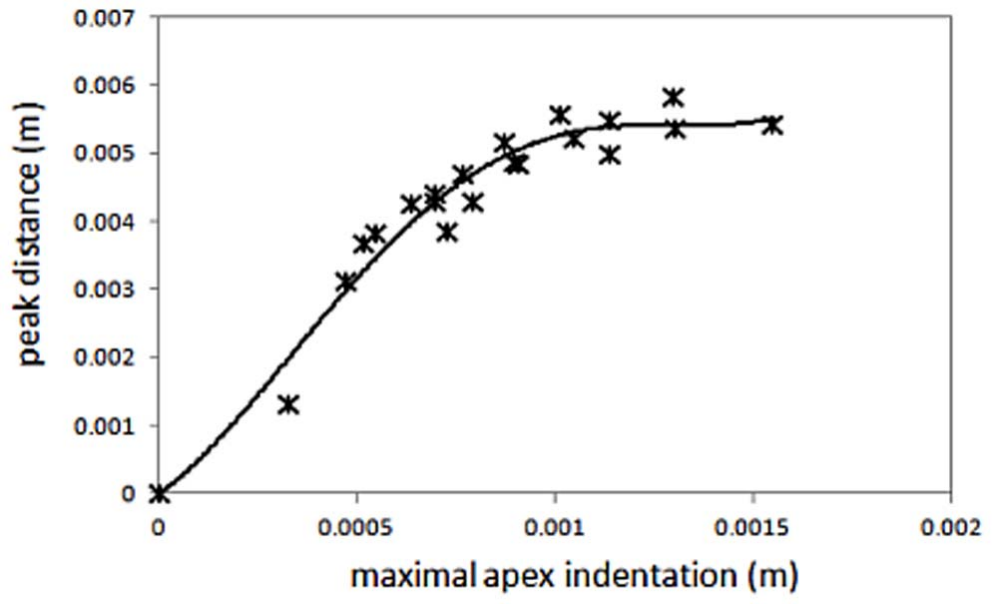
Correlation between the peak distance and apex indentation obtained from an ample experimental data set, including corneal response under different stiffness, thickness and IOPs.

**Figure 3:**
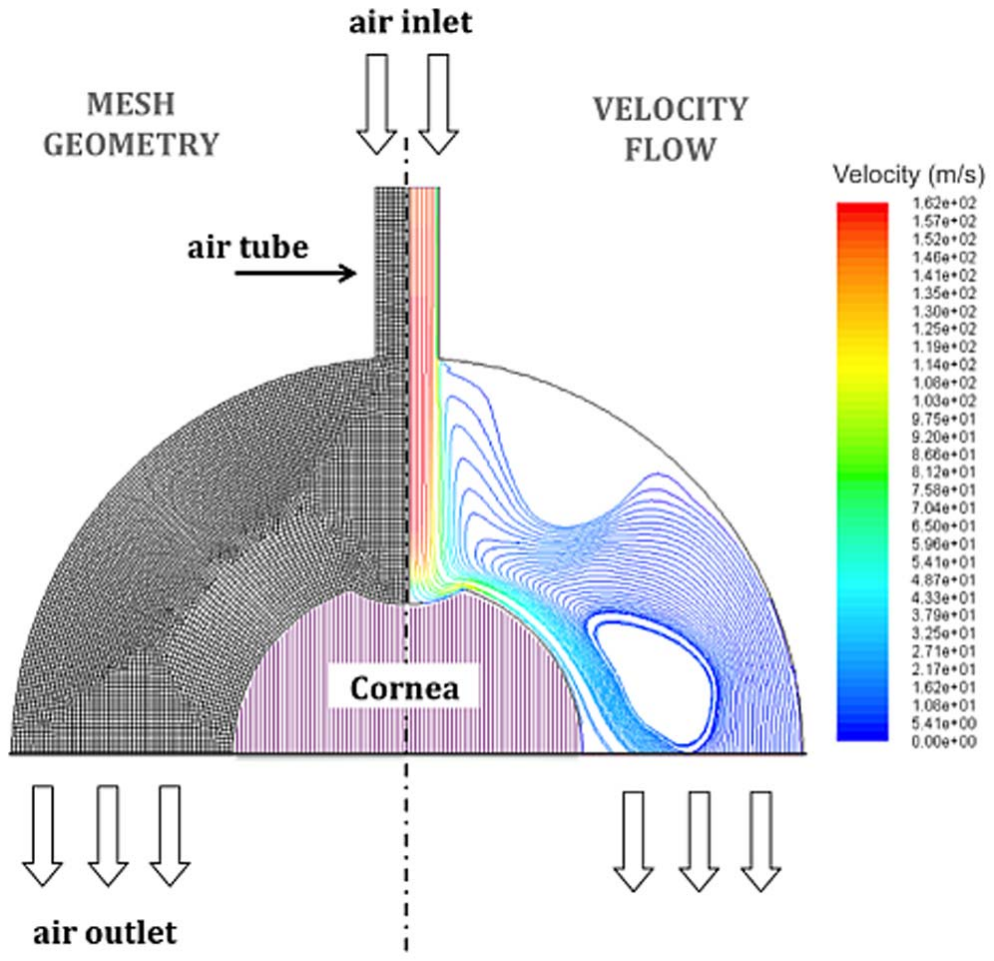
Geometry model of the cornea at maximal deformation. Mesh of cells of the modeled air volume (left) and streamlines (right) colored by the flow velocity distribution.

Prior experimental data show a correlation between the maximum deformation amplitude of the cornea and the peak-to-peak distance. Figure 2 shows a polynomial fit between these two deformation parameters and indicates that the deformed corneal geometries can be parameterized using maximum corneal indentation as a free parameter. For the current model, six representative positions were sampled. Figure 3 shows the mesh of the modeled air volume for the cornea in its maximal deformed state, along with the resulting airflow velocity distribution. The geometrical parameters of the meshes for the six different corneal geometries are shown in Table 1.

**Table 1:** Geometrical parameters of deformed corneas and the corresponding mesh size.

Case n°	Apex indentation[mm]	Peak distance[mm]	Number of cells in mesh	Average cell size[mm^2^]
0	0	0	5600	0.0464
1	0.5	3	5754	0.0452
2	0.6	4	5703	0.0457
3	0.8	5	5858	0.0446
4	1	5.3	5858	0.0446
5	1.5	5.5	6925	0.0378

#### CFD Boundary conditions

The air-puff emitting tube and the cornea were considered as wall type boundary conditions (i.e. the cornea was simulated as a rigid body not considering fluid-structure interaction). A velocity inlet was modeled at the end of the air tube and a pressure outlet around the cornea. The initial flow velocity of the air-puff (115 m/s) was calculated from the stagnation pressure, i.e. the peak central pressure measured in the air-puff characterization. As inertial effects are expected to dominate over viscous effects, turbulent flow was assumed and described using the Reynolds stress model. The Reynolds averaged momentum equations for the mean velocity are:
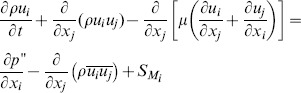
(2)where 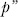 is a modified pressure, 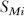 is the sum of body forces and 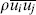 corresponds to the fluctuating Reynolds stress contribution. Unlike eddy viscosity models, the modified pressure has no turbulence contribution and it is related to the static (thermodynamic) pressure by:
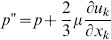
(3)


In the differential stress model, 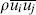 is made to satisfy a transport equation. A separate transport equation must be solved for each of the six Reynolds stress components of 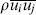. The differential equation Reynolds stress transport is:
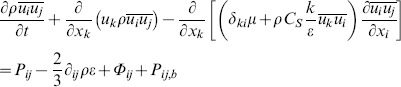
(4)where 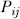 and 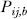 are shear and buoyancy turbulence production terms of the Reynolds stresses respectively, 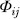 is the pressure-strain tensor, and 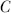 is a constant.

**Figure 4:**
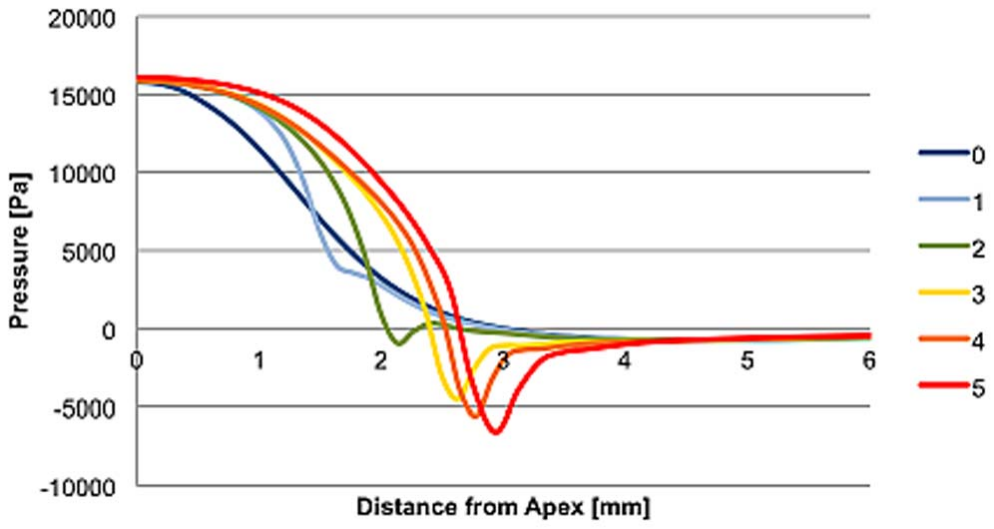
Spatial pressure distribution of the air-puff along the corneal surface for different deformed shapes.

**Figure 5:**
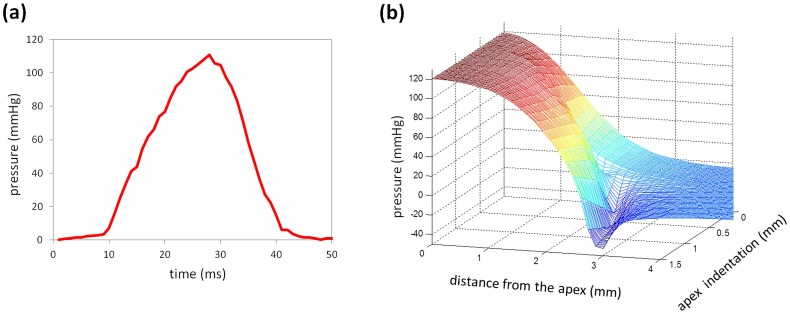
Air-puff characterization. (a) Experimentally measured temporal air-puff profile; (b) Results from CFD simulation showing the air-puff as a function of apex indentation and location along the cornea (horizontal distance from the apex).

The pressure distribution along the corneal surface was obtained for each of the six deformed corneal geometries. Figure 4 presents the resulting spatial pressure profiles for different corneal apex indentations, which were then interpolated (with an indentation step-width of 20 µm) into a 2D spatial pressure surface and used as an input in further analysis. The shape of the pressure distribution did not change significantly with varying flow speed, but it was altered as the cornea deforms (see Figure 5b).

In order to account for measurement inaccuracies of the maximal pressure and hence the estimation of the air speed the spatial pressure function was normalized and a scaling factor P_max_ introduced. This was necessary as the sensor, due to the geometry of its housing, tends to overestimate the pressure in high-speed dynamic measurements. In the structural finite element simulations, the applied surface pressure was a function of apex indentation, distance from the apex and time.

### Structural finite element simulation

#### Geometry

A 2D axis-symmetric eye model was defined corresponding to the different corneal conditions and boundaries (see Table 2 for parameter details of the eye model). The outer coat of a half eye globe was considered – consisting of cornea, limbus and sclera – and modeled by 8-node elements with quadratic displacement behavior. Thereby 400 elements were used to represent the cornea (where corneal thickness was divided into 8 elements), 56 elements to represent the limbus, and 640 elements to represent the sclera. Initial corneal curvatures were adjusted to match the experimental values after IOP (15 mmHg) application. Scleral geometry was taken from the literature [33] and the limbus was defined by connecting cornea and sclera. The ocular humors were modeled by a single fluid compartment, which consisted of 137 hydrostatic fluid elements.

**Table 2:** Biomechanical and geometrical model parameters used to simulate the human and pig corneal deformation.

	Human (virgin)	Porcine (cross-linked)
Corneal thickness (µm)	558	211
Anterior curvature (mm)	8.03	8.06
Posterior curvature (mm)	6.86	7.54
Corneal diameter (mm)	10	12
Scleral diameter (mm)	19.5	23.5

#### Material models

The corneal tissue was modeled by a linear viscoelastic material. Thereby only the shear response was considered, as it is typically dominant over the volumetric response.
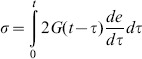
(5)where 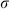 is the Cauchy stress, 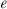 the deviatoric strain and 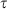 past time, which was described by a two parameter Prony series:



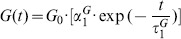
(6)with 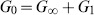 and 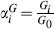 where 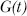 is the Prony shear modulus, 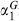
is the relative modulus, 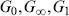 are the instantaneous, the infinite and the current shear elastic moduli, respectively and 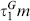
the relaxation times for the Prony component. The shear modulus (G) denomination was only used to express viscoelasticity. In order to compare to the commonly used elasticity modulus (E), we used the following equation for isotropic materials 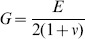 where 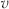 is the Poisson’s ratio. Limbus and sclera were described by a purely elastic material: 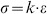, where 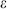 represents strain. It should be noted that these material models represent the macroscopic response of the biological tissues. The microscopic structure was not considered for the relatively small strains present during the deformation. Instead, the cornea was divided in two equal layers (anterior and posterior, extending 50% of the entire corneal depth each) and a different elastic modulus was assigned to each, based on the considerable differences in the morphology of the anterior and posterior cornea (collagen interweaving, susceptibility to swelling, mechanical structure [34]) that have been reported in the literature [16], [35] in both, virgin and cross-linked corneas. In fact, changes in the elasticity across the corneal depth are likely gradual [16], but the definition of two layers with each of them representing the mean corneal stiffness in the anterior or posterior cornea respectively, allows capturing the physiological reality while keeping the number of variables suitable for adequate convergence of the optimization routines. The aqueous and the vitreous humors were defined as a hydrostatic fluid with a pressure equal to the IOP. Fluid-structure interaction was considered between the ocular tissues and the ocular humors by relating the solid stress and displacements to the pressure imposed on the interface by the fluid pressure load (neglecting friction):

Then the fluid pressure load vector 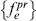
was added to the basic equation of motion,
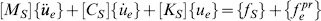
(7)where 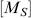 is the structural mass matrix, 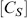 the structural damping matrix, 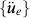 the elemental acceleration vector, 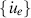
the elemental velocity vector, 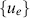
the elemental displacement vector and 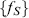 the structural load vector.

Biomechanical parameters of the sclera and limbus (see Table 2) were defined by data obtained from the literature [36]. The corneal density was set to [37] ρnormal = 1062 kg/m^3^ for a 700 µm corneal thickness and scaled according to the thickness values in the experiments. A material damping of 10 µs was used.

#### Boundary conditions and loads

For the simulations of the ex vivo whole globe the sclera was fixed mimicking the fixation of the eye in the eye-holder in the experiments. For the in vivo condition the eye globe was damped along on the vertical symmetry axis representing the ocular muscles and other surrounding fatty tissue. It was assumed that those external damping factors can be summarized in a single vertical damping element, while horizontal damping effects were neglected. The vertical damping was implemented by a mass-less longitudinal spring-damper, which was modeled by a uniaxial tension-compression element, defined by a spring constant (5·10^6^ N/m) and a damping coefficient (1.0). The intraocular pressure was applied in the model to the interior surfaces of cornea, limbus and sclera according to the experimental data (15, 20, 25 and 35 mmHg).

#### Air-puff application

A pressure load was applied on the element edges of the anterior corneal surface according to the spatial pressure distribution of the air-puff at the different indentation depths. This was necessary because the fluid dynamics characteristics change significantly as the cornea deforms. Furthermore the pressure variation with time (measured experimentally with the pressure sensor) was considered by multiplying the current pressure with the normalized temporal pressure profile.

#### Load steps

In the first load step the IOP was applied to the model. Then, in the next step the load modeling the air-puff was applied. In order to achieve sufficient temporal resolution, this step was divided into 49 sub-steps, so the applied pressure was updated every 625 µs.

### Multi-step optimization

**Figure 6:**
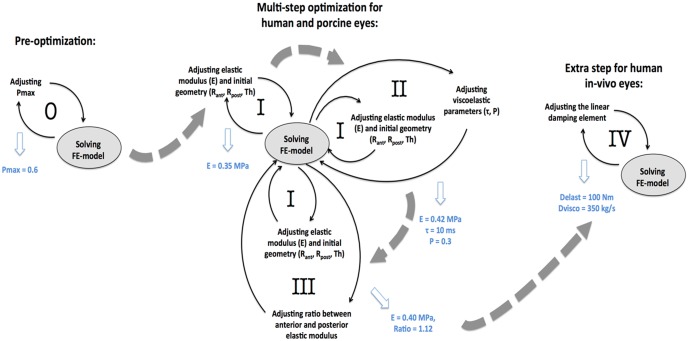
Schematic of the optimization process. Pre-optimization step (0), where the maximal air-puff pressure is adjusted; Multi-step optimization, comprising adjustment of initial elastic modulus and corneal geometry (I); adjustment of viscoelastic parameters (II) and further refinement of the elastic modulus and geometry (I); adjustment of the anterior and posterior elastic moduli (III), followed by a further refinement of the elastic moduli and geometry (I). In human eyes and additional step was incorporated to account for damping by ocular muscles and external tissue (IV). In the illustration the size of the loops is positively correlated with the dominance of the parameter adjusted therein. The resulting parameter values, shown in blue, represent an example of the output parameters at each step for human eyes ex vivo and in vivo.

**Figure 7:**
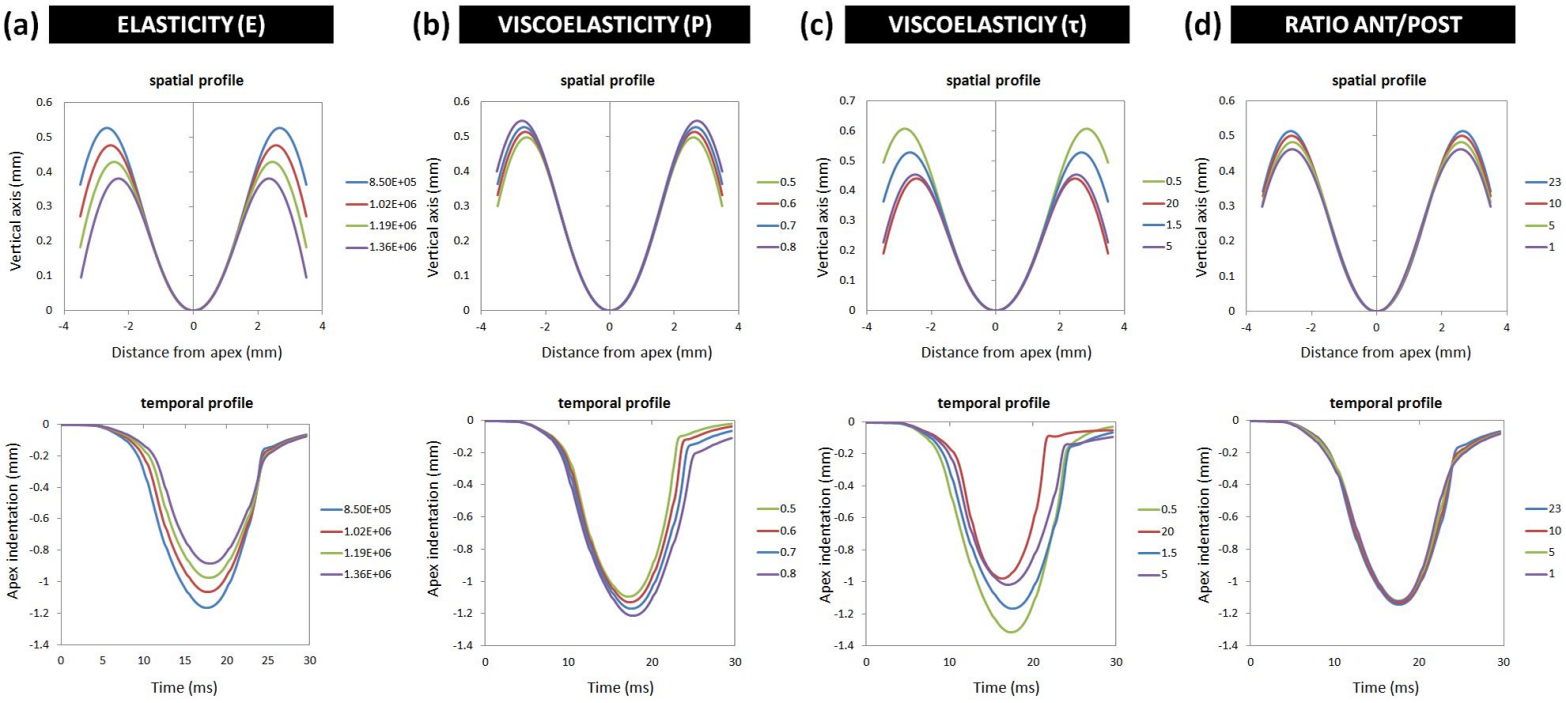
Effect of the change of different biomechanical parameters on the spatial deformation profiles (upper row) and temporal deformation profiles (lower row) at IOP = 15 mmHg. (a) Elastic properties dominate the maximal indentation depth. (b) Viscoelastic properties dominate the amount of hysteresis when the air-pressure has decreased to zero. (c) The ratio between anterior and posterior stiffness dominates the distance between corneal apex and bending points.

A multiple step optimization approach (schematic shown in Figure 6) was applied to find the biomechanical parameter set that best matched the experimentally observed behavior. Thereby in each step, the corresponding parameters were first scanned within their limits and then further optimized by a gradient-based approach. Generally, the spatial profile is dominated by the elastic properties, while the recovery part of the temporal deformation profile is dominated by the viscoelastic properties (see A, B in Figure 7). For each step, the deformed geometry obtained from the FE-simulation was exported, analyzed in terms of the temporal and spatial deformation profile and compared to the experimental data.

In the beginning, the elasticity modulus was the only variable considered to account for the deformation behavior. Then the biomechanical description of the material model was refined in consecutive steps, adding viscoelastic properties and a difference between anterior and posterior corneal stiffness. Thereby the fact that two deformation profiles (temporal, spatial) were available, and that the optimization variables had differential effects on each, reduced the amount of possible local minima. The multi-step procedure allowed us to obtain better comparable parameter sets across the different tested conditions.

#### Pattern of the cost-function for the different optimization parameters

In order to define the individual steps of the optimization procedure, we studied the pattern of influence for each optimization variable on the temporal and on the spatial corneal deformation profiles. Changing a given variable subsequently, while keeping the other parameters constant allowed then identifying systematic effects.

Figure 7 shows the result of this analysis. We found that (a) increasing the elastic modulus decreases the maximal corneal indentation depth and leads to a delayed indentation in the temporal profile; (b) increasing the Prony constant has little impact on the spatial profile, but increases the hysteresis in the temporal profile; (c) decreasing the time constant τ increases the amount of corneal indentation and determines the hysteresis and duration of the deformation event in the temporal profile; (d) increasing the stiffness in the anterior region of the cornea with respect to the posterior region has a little impact on the temporal profile, but increases the vertical distance between corneal bending points and corneal apex, especially at higher IOPs.

#### Pre-optimization

The different patterns were subsequently used to optimize the geometrical and biomechanical parameter set of the FE-model, as illustrated in Figure 6. In a step prior to the optimization, P_max_ was adjusted in the range from 65.8 mmHg (115 m/s) to 111 mmHg (156 m/s) using the deformation profiles of the cross-linked porcine cornea at 15, 20, 25 and 35 mmHg. This led to a value of P_max_ = 72.5 mmHg, which was then kept constant for all conditions (see “pre-optimization” in Figure 6).

#### Optimization

In the first step of the optimization a very simplified material model - linear elasticity - was assumed. It included the adjustment of geometrical parameters and the elastic modulus: the initial anterior radius of curvature, central corneal thickness and elastic modulus were adjusted so that after applying the intraocular pressure, both the modeled and experimental corneal geometry within the 8-mm diameter central zone and the maximal indentation depth were identical.

In the second step the material model was refined adding viscoelastic properties expressed by a Prony and a relaxation time constants. This second step was iteratively coupled with the first step in order to correct for the viscoelastic component, the effects of which were initially accounted for by the previously adjusted elastic modulus (see central part in Figure 6). The viscoelastic parameters were adjusted in order to reproduce the hysteresis in the temporal deformation profile, which is observed after the air-puff stops, i.e. in the zone of indirect air-puff response.

The third step provides a further refinement the model accounting for physiological differences in the anterior and posterior collagen interweaving, which result in an in-depth variation of corneal stiffness. This difference in stiffness between the anterior and posterior corneal regions was introduced in order to adjust the spatial corneal deformation profile (see Figure 7 c). In normally hydrated corneas the anterior cornea is approximately 16% more rigid. In cross-linked corneas the difference between the anterior and posterior corneal stiffness is larger, as typically only the 60% of the entire cornea is stiffened after treatment [38].

For the in-vivo human cornea a fourth step was necessary, which included the adjustment of the two elements (elastic and viscous parameters) of the external damping element (see “extra step for in vivo eyes” in Figure 6).

### Sensitivity Analysis

After selecting the model parameter set that best represented the virgin human cornea in vivo condition, a sensitivity analysis was performed in order to determine the geometrical and mechanical parameters that dominate the shape and amount of corneal deformation following the air-puff. Seven parameters were selected (corneal thickness, stiffness, curvature, density, IOP and two viscoelastic constants) and changed consecutively within physiological or pathological ranges. For each parameter variation the effect on the overall predicted corneal deformation was determined by analyzing the coordinates of the deformed shape, including changes in the maximal corneal indentation and peak distance. Evaluation of the factors that dominate the corneal deformation is highly relevant in the clinical practice, where diagnostics is currently performed from geometrical deformation parameters.

### Computing techniques

ANSYS APDL structural mechanics code (ANSYS, Inc., Canonsburg, PA) was used for the mechanical simulations and the FLUENT module for the CFD simulations. The analysis of the deformed corneal shape was performed in Matlab (The MathWorks, Natick, MA).

## Results

### Air-puff modeling

Figure 5 (a) depicts the experimentally measured temporal pressure profile at the center of the air-puff. A maximal air pressure of 120 mmHg at the corneal surface was present. The geometry-dependent spatial pressure profile was obtained from a separate computational fluid dynamics (CFD) simulation. Figure 5 (b) shows the resulting spatial air-pressure profile expressed as a function of maximal corneal indentation at the apex and distance from the apex.

### Corneal response simulation under different conditions

The finite element model could well reproduce average experimental data from a previous study [21] of corneas under different intraocular pressures (IOPs) and following collagen cross-linking.

#### Corneal deformation for different intraocular pressures (IOPs) – porcine eye model

**Figure 8:**
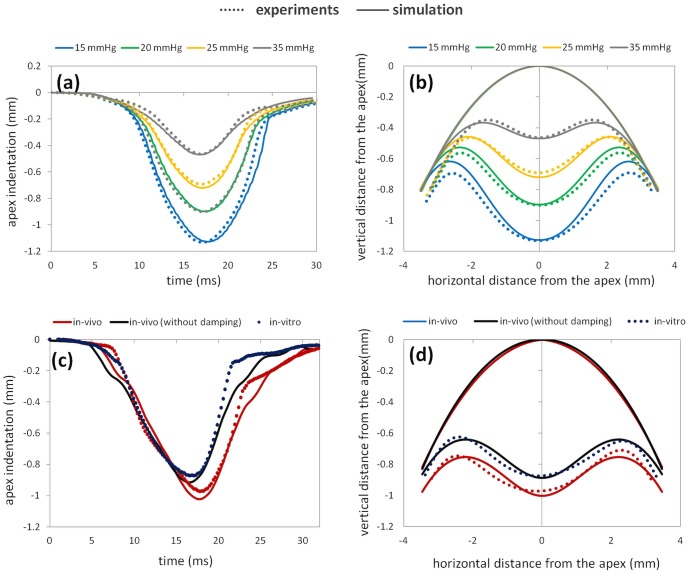
Temporal (a, c) and spatial (c, d) corneal deformation with air-puff. Dotted lines represent experimental corneal deformations and continuous lines simulated corneal deformations Panels (a, b) show data for porcine corneas: simulated and experimental data at different IOPs. Panels (c, d) show data for human corneas: simulated response with and without ocular muscle damping, compared to in vivo experimental deformations measured in patients and ex vivo deformations measured in an enucleated whole globe.

The corneal response upon air-puff ejection was simulated for different IOPs (ranging from 15 to 35 mmHg, i.e. covering the IOP range from physiological values to those found in severe glaucoma). We found that the maximum corneal apex indentation was 1.13 mm for the lowest IOP and 0.46 mm for the highest IOP. Air-puff maximum pressure and, to a lesser extent, differences in stiffness between the anterior and posterior cornea were found to play a major role in the predicted corneal deformation. Figure 8 a, b shows the simulated corneal deformation compared to experimental corneal deformation^21^ in cross-linked porcine corneas ex vivo for different IOPs. The reconstructed elasticity moduli from the model were: E_anterior_ = 25.5 MPa; E_posterior_ = 0.85 MPa (see Table 3). Both the decreased corneal apex indentation (Δ_fem_−0.676 mm versus Δ_exp_−0.666 mm) with increased IOP from 15 to 35 mmHg and the decreased peak distance, i.e. horizontal distance between the corneal bending points at maximal deformation (Δ_fem_−2.04 mm versus Δ_exp_−2.34 mm) are well reproduced by the model.

**Table 3:** Corneal biomechanical parameters obtained from finite element analysis: Elasticity modulus represents the static material properties; The difference between anterior and posterior cornea describes the differences in collagen interweaving and the resulting higher anterior corneal rigidity; The relaxation time – which must lie within the temporal scale that was analyzed – describes the point of time at which the corneal stiffness decreased by the factor of the relative modulus; The muscle spring and damping constants describe the static and dynamic displacement, respectively, due to whole the eye movement in in vivo measurements.

	Human	Porcine
Condition	virgin	cross-linked
Elasticity modulus (Mpa)posterior cornea	0.40	0.85
Difference between anterior and posterior	1.12	30
Relaxation time (ms)	10	1.5
Relative modulus	0.3	0.7
Muscle spring constant (Nm)	350	-
Muscle damping constant (kg/s)	100	-

The latter two parameters represent accumulated contributions of muscles and fatty tissue and were not further interpreted.

The relation between corneal indentation and eye globe compression (i.e. general deformation from a spherical to an elliptical eye shape resulting from scleral deformation) depended on the IOP, but also on the difference between the anterior and posterior corneal stiffness. At 15 mmHg, corneal deformation contributed 90.4% to the overall indentation, while at 35 mmHg only 44.9%. We also found that increasing the difference between anterior and posterior corneal stiffness produced larger corneal deformation (i.e. for E_anterior_/E_posterior_ = 30: corneal deformation = 90.4%; for E_anterior_/E_posterior_ = 1: corneal deformation = 83.7%).

The viscoelastic effect was evidenced by the remaining deformation (between 18.7 and 24.1% of the overall deformation at different IOPs), which gradually decreased with time after the air-puff event. The viscoelasticity of the cross-linked porcine corneas ex vivo was described by a relative modulus of 0.7 and a relaxation time constant of 1.5 ms, and contributed with 46% to the maximal apex indentation.

#### Corneal deformation in vivo – human eye model

Defining a damping element for the fixation of the ocular globe allowed us to describe the effect of ocular muscles and fatty tissue. Corneal deformation in vivo (average across 9 eyes) was compared to simulations where damping of the entire ocular globe was considered, while corneal deformation ex vivo was compared to simulations without damping (see Figure 8 c, d).

We found that the retro-bulbar tissue contributed 0.112 mm to the apex displacement and induced a hysteresis (additional to the viscoelastic hysteresis of the cornea) in the temporal profile in the region of indirect air-puff response.

### Retrieved corneal biomechanical parameters

Table 3 summarizes the viscoelastic parameter set retrieved for the porcine and human cornea. The retrieved corneal biomechanical parameters varied across different treatments and conditions. In virgin human corneas, the differences in anterior and posterior corneal stiffness are consistent with recently reported corneal stiffness gradient [35]. In cross-linked porcine corneas the difference between anterior and posterior cornea was 26.8 times higher than in virgin corneas, consistent with the stiffening effect of the cross-linking occurring in the anterior cornea.

### What determines the corneal response?

A sensitivity analysis was performed for the human eye in vivo (i.e. with damping) in order to evaluate which geometrical and biomechanical parameters determine the corneal deformation with an air-puff. Table 4 presents the predicted dependency (gradients) of the apex indentation and peak distance (distance between the highest lateral points of the cornea at maximal deformation) on different geometrical and biomechanical parameters. Dependencies on absolute parameter variations were also investigated. Table 4 represents data for parameter variations within the estimated physiological range. The sclera also had a significant effect, increasing the apex indentation by 0.20 mm when changing its rigidity by 90%.

**Table 4:** Parameter gradient from the sensitivity analysis.

Geometrical/biomechanical parameters	Change within the physiologic range	Δ peak distance (mm)	Δ apex indentation (mm)
Thickness (mm)	Δ 100 µm	−0.7168	0.2011
Stiffness (MPa)	Δ 0.2 MPa	−0.7218	0.3764
Relative modulus (no unit)	Δ 1	0.8929	0.1000
Relaxation time (ms)	Δ 10 ms	0.4300	−0.2324
IOP (mmHg)	Δ 40 mmHg	−2.472	0.2320
Curvature (mm)	Δ 1 mm	0.1875	−0.0163
Density (10^6^g/m3)	Δ 500 kg/m3	0.0008	0.0000

The table lists changes in the peak distance and apex indentation for parameter (thickness, stiffness, relative modulus, relaxation time, IOP, curvature, density) for values within the expected physiological/pathological ranges.

## Discussion and Conclusions

New imaging acquisition techniques allow capturing the dynamic geometrical deformation response of the cornea following an air-puff. To our knowledge, we have presented for the first time numerical estimates of corneal elastic and viscoelastic parameters, based on Scheimpflug imaging of the spatio-temporal dynamic corneal deformation and sophisticated Finite Element Modeling. The simulation reproduces with high accuracy the corneal deformation patterns, while the estimated biomechanical parameters are independent of geometry. Overall, cross-linked porcine corneas were 9.38 times more rigid than virgin human corneas. The much higher stiffness of the anterior cornea than of the posterior in cross-linked corneas can be primarily attributed to the cross-linking treatment - literature reports an increase in corneal stiffness between 72% and 329% after CXL [39] - but also to the dehydration and hence a higher density in the anterior corneal region produced by the photosensitizer solution. In a recent publication [9] we showed that the corneal hydration state affects its biomechanical response.

In addition, by simulating different boundary conditions, the movement of the cornea in response to the air puff could be isolated from the additive effects of scleral deformation and movement of the whole eye observed in the Scheimpflug images.

The dynamic response [8], [10] of a material can be very different from its static [7] behavior, as viscoelastic materials typically behave more rigid the faster a loading condition is applied [40]. Dynamic analysis hence will provide information on the instant rigidity of a material, while static material properties represent the elasticity at infinity. Dynamic properties of corneal tissue are likely dominated by the extracellular matrix, while static properties give information on the collagen structure. Combining static and dynamic analysis therefore might allow a better understanding of the interaction between the extracellular matrix with the collagen structure. The air-puff corneal imaging technique addresses dynamic properties of tissue in a similar time range (20 ms) as ultrasound-based elastography (50 MHz) and magnetic resonance imaging (300 MHz) and in a longer time range than Brillouin microscopy (GHz). Thereby the new technique surpasses the first two in patient comfort, the second two in acquisition rates, and allows retrieving corneal viscoelasticity and elasticity. Mean corneal Young’s modulus as determined in this study from air-puff deformation was 0.71 MPa for the virgin human cornea and 13.2 MPa for the cross-linked porcine cornea. These values fall within the range of corneal stiffness reported from ultrasound elastography ranging from 0.19 MPa to 20.0 MPa [41], [42]. Young’s moduli obtained from magnetic resonance imaging were lower (0.04 to 0.19 MPa) [12], suggesting that the time range in which the measurements are acquired (ultrasound and air-puff measurements are performed more rapidly than magnetic resonance measurements) play an important role in the observed stiffness of the corneal tissue. Further factors that might affect the experimental assessment of corneal stiffness include the post-mortem time and the tissue hydration [9].

Time scales are not only relevant when comparing different measurement systems, but also in regard to the physiological interpretation. Elastic properties, i.e. static properties, are determined by the collagen matrix and depend on the microstructural arrangement as well as the number of cross-links. These material properties determine the long-term corneal resistance against the IOP. Viscoelastic properties, i.e. time-dependent properties, describe the rearrangement of the extracellular matrix (mainly due to diffusion of water) upon changes in stress. Generally the shorter the time scale where material properties are analyzed, the harder the material and the larger the viscoelastic contribution. Thereby the time scale of interest for corneal material properties depends on the time scale of the treatment or pathology: For refractive surgery short-term viscoelasticity is probably more important, while in keratoconus long-term viscoelasticity is of interest. Future air-puff systems may be provided with alternatives to allow a viscoelastic analysis at different time scales.

Although the sensitivity analysis showed a correlation between corneal rigidity and corneal deformation, geometrical parameters, such as the central corneal thickness, as well as IOP, also showed a large effect. This means that all factors need to be considered and entered as input parameters in the simulation, which increases the complexity of the finite element simulation. Comparison of the gradients obtained from sensitivity analysis with clinical data - based on thickness changes after LASIK surgery, and over a physiological IOP range (personal communication by Oculus) - show good agreement.

Although the multi-step optimization approach has allowed a systematic retrieval of the model parameters, a potential limitation of this method might be the identification of a local rather than a global minimum. Nevertheless, the characteristic pattern how individual parameters changed the cost-function as well as the fact that a single parameter set (CXL porcine corneas) allowed an accurate reproduction of the corneal response at different IOPs (15–35 mmHg), suggesting that the solution is robust and likely to be unique.

A potentially strong limitation of this study is that linear material properties were assumed. Experimental diagrams from uniaxial stress-strain testing suggest a linear stress-strain relation up to about 5–6% strain [39]. Although most regions of the cornea presented less than 6% strain (after IOP application and during the air-puff event), maximal strains at the apex were up to 10%, slightly over the linear range. This may have led to inaccuracies in the regions of maximal bending resulting in a slightly different overall deformation response. A simplification in the model is the assumption of axis-symmetry, which does not capture potential differences in the horizontal and vertical meridian. A 3D expansion of the model would allow incorporating asymmetries such as those occurring in keratoconus, but at the same time it would require acquisition of spatial corneal deformation profiles in multiple meridians. An assumption of this study was that changes in the mechanical response of the cornea could be described by the parameters listed in Table 3. Although more parameters generally allow a more detailed description, when comparing different conditions it is important to have unique parameter sets. These typically only can be obtained using a limited number of optimization parameters.

We believe that finite element modeling is an extremely valuable tool for the analysis of in vivo dynamic corneal deformation. Inverse simulation together with state of the art imaging systems will allow the analysis of (in particular the viscoelastic) corneal biomechanical properties (viscoelastic properties in particular) in a clinical setting, facilitating diagnosis of corneal pathologies, patient screening for refractive surgery and evaluation of treatment efficacy such as after cross-linking.
